# Job demands, job resources, and health outcomes among nursing professionals in private and public healthcare sectors in Sweden – a prospective study

**DOI:** 10.1186/s12912-022-00924-z

**Published:** 2022-06-06

**Authors:** Dip Raj Thapa, Johanna Stengård, Anette Ekström-Bergström, Kristina Areskoug Josefsson, Alexandra Krettek, Anna Nyberg

**Affiliations:** 1 Department of Nursing and Reproductive, Perinatal and Sexual Health, School of Health Sciences, University of Skövde, PO Box 408, 541 28 Skövde, Sweden; 2 School of Health and Welfare, Jönköping University, Box 1026, 551 11 Jönköping, Sweden; 3 Department of Psychology, Stress Research Institute, Stockholm University, 106 91 Stockholm, Sweden; 4 Department of Health Sciences, University West, Gustava Melins gata 2, 461 32 Trollhättan, Sweden; 5 Department of Behavioural Sciences, Faculty of Health Sciences, Oslo Metropolitan University, PO Box 4, 0130 Oslo, Norway; 6 Faculty of Health Studies, VID Specialized University, Vågsgaten 40, 4306 Sandnes, Norway; 7 Department of Public Health, School of Health Sciences, University of Skövde, PO Box 408, 541 28 Skövde, Sweden; 8 Department of Internal Medicine and Clinical Nutrition, Institute of Medicine, Sahlgrenska Academy at University of Gothenburg, Box 400, 405 30 Gothenburg, Sweden; 9 Department of Community Medicine, Faculty of Health Sciences, UiT The Arctic University of Norway, PO Box 6050, 9037 Langnes, Norway; 10 Department of Public Health and Caring Sciences BMC, Uppsala University, Husargatan 3, Box 564, 751 22 Uppsala, Sweden

**Keywords:** JD-R model, Occupational health, Demands, Resources, Healthcare, Employment sectors

## Abstract

**Background:**

Nursing professionals exhibit high prevalence of stress-related health problems. Job demands and job resources are parallel drivers of health and well-being among employees. Better job resources associate with better job satisfaction, job motivation and engagement even when job demands are high. To date, there is limited research which explores the association between job demands, job resources and health outcomes among nursing professionals in the Swedish context. The aim of this study was therefore to investigate Swedish nursing professionals’ job demands and job resources in relation to health outcomes, with comparisons between the private and public healthcare sectors. The specific research questions were as follows: (1) Are there differences between private and public healthcare regarding job demands, job resources, and health outcomes? and (2) Are there prospective associations between job demands and job resources in relation to health outcomes?

**Methods:**

Data were drawn from the Swedish Longitudinal Occupational Survey of Health (SLOSH) 2016 and 2018, including 520 nurses and 544 assistant nurses working in the private and public healthcare sectors from 2016 (baseline). Data were analyzed using binary logistic regression.

**Results:**

Nursing professionals reported higher threats, lower bullying, lower control, lower social support, and lower cohesion in the public healthcare units compared to the private healthcare units. The prospective analyses showed that job resources in terms of social support and rewards were associated with higher self-rated health and lower burnout. Cohesion was associated with higher self-rated health. Job demands in terms of psychological demands and job efforts were associated with lower self-rated health, higher burnout, and higher sickness absence, while emotional demands were associated with higher burnout.

**Conclusions:**

Nursing professionals’ job resources are deficient in public healthcare units. Job resources are associated with positive health outcomes, whereas job demands are associated with negative health outcomes, among nursing professionals. Strengthening job resources among nursing professionals in the private and public healthcare sectors can promote and sustain their work-related health.

## Background

Work-related health challenges and adverse health outcomes, such as musculoskeletal disorders, stress, and burnout, are increasing within occupational health worldwide [1, 2]. Within the European Union, work-related stress adversely impacts workers’ health and adds economic burden to the society [2]. Nordic countries, including Sweden, exhibit the highest proportion of employees who report psychosocial health problems in the workplace [3]. In Sweden, sick leave has increased continuously from 2010, particularly among women in human service organizations [4]. In 2018, this trend stabilized, as long-term sickness absence started to decrease and differences in sickness absence between men and women were minimized [5]. Healthcare organizations in Sweden have poor organizational and psychosocial work environments in comparison with other organizations [6].

Although healthcare systems differ between European countries, they share common reasons for burnout, which are related to organizational and psychosocial workplace factors [7]. Besides organizational challenges, the nursing profession is confronted by increasing professional demands and the growing needs of an aging population [8, 9]. In addition, there is a global shortage of nursing professionals [10, 11].

The global prevalence of burnout among nursing professionals, e.g., midwives [12], nurses [13], and assistant nurses (nursing staff) [14], is evident in the results of literature reviews and meta-analyses. Burnout and sickness absence among employees within human service occupations are common [15]. Vulnerability in the nursing profession arises from psychosocial health problems, organizational demands, and care-related demands, such as moral distress at work [16, 17]. Experiencing threats and violence [18], multiple responsibilities, high job demands, and low job resources [19, 20] are also common within the nursing profession. Indeed, high job demands combined with low job resources are associated with long-term sickness absence [21] and illnesses such as burnout and depression [20, 22].

Although its demands are high and there is a high prevalence of stress and burnout, the nursing profession is associated with high work engagement [23, 24]. Without the physical involvement, cognitive alertness, and emotional connection of health professionals, especially nurses, patients could not be provided with sufficient quality of care [25]. Therefore, it is crucial for the health outcomes of health professionals that they have ample job resources to handle the significant job demands they face. This is because high job demands in combination with sufficient job resources can increase job motivation and work engagement [26].

### Job demands-resources model

The job demands-resources (JD-R) model [27, 28] is a universal model that to date has been applied worldwide in different populations. The two distinct categories of the JD-R model, job demands and job resources, are the driving forces behind the well-being of employees. Job demands refer to organizational characteristics that require physical and psychological (cognitive and emotional) efforts, which may lead to adverse health outcomes, such as burnout [27, 29]. Job resources refer to aspects of the work environment that contribute to personal growth and development. Such resources enable the employee to achieve work goals, which in turn results in positive health outcomes and increased motivation [27, 29]. In the nursing profession, a higher level of physical, cognitive, and emotional demands is associated with a lower level of work engagement [30] and reduced quality of care for patients [31]. Another important factor associated with nursing personnel’s job demands and job resources is the ownership and administration of private and public healthcare sectors [32].

### Organizational changes and their impact on employees’ health

The impact of privatization on employees’ health is complex and varies by country. For instance, employees’ health is better in the private than in the public healthcare sectors in Jordan [33], South Africa [34], and Spain [35], and worse in Greece [36], Taiwan [37], and the United Kingdom [38].

The Swedish healthcare system is based on principles that are equally applied to private and public healthcare sectors. A continuous privatization of the healthcare sector has occurred in Sweden since 2011, especially primary care [39]. At present, approximately 20% of all healthcare is provided by the private healthcare sector [40]. Falkenberg, Näswall [41] found that employees in the private healthcare sector had lower job motivation than employees in the public healthcare sector, whereas a recent report by the Swedish Social Insurance Agency [4] concluded that differences regarding employees’ sickness absence and type of employment sector were minimal. However, possible differences in work environment and employees’ health between the private and public healthcare sectors in Sweden are to date largely unknown.

The association between job demands, job resources and health outcomes has been established in previous research of the general work population as well as in nursing professionals. For example, high job demands have been found associated with long-term mental disorders and sickness absence in the Swedish Twin study [21] and in Malaysian employees, high physical and emotional job demands were associated with burnout and sleep problems [42]. In contrast, social support has been found to increase nursing staff’s self-efficacy and to provide a sense of security [43]. However, there is limited research on job demands and resources of nursing professionals in the Swedish workforce. A better understanding of how demands and resources influence nursing professionals’ health outcomes in the private and public healthcare sectors is critical to sustain and promote their health.

### Aim

The aim of this study was to investigate Swedish nursing professionals’ job demands and job resources in relation to health outcomes, with comparisons between private and public healthcare sectors. The following research questions were addressed: (1) Are there differences between private and public healthcare regarding job demands, job resources, and health outcomes? and (2) Are there prospective associations between job demands and job resources in relation to health outcomes?

## Methods

### Study design and setting

This study has a prospective design and was carried out in Sweden using a questionnaire survey. The study included nursing professionals from both private and public healthcare sectors. The public healthcare sector included employees from municipalities and county councils. The municipalities included employees only from municipality care, while the county councils included employees from both primary and hospital care. Sweden’s healthcare system is organized into local, regional, and national levels. Four regional bodies and 17 county councils are responsible for healthcare services for their respective populations [39]. County councils are responsible for primary healthcare as well as hospital and specialized healthcare, and municipalities are responsible for the care and housing needs of elderly and disabled people in their demographic area [39]. Private healthcare is divided into two categories: (1) healthcare services under contract with county councils, local authorities, and municipalities; and (2) healthcare services with no contract with the public healthcare system [44]. Since 2010, private healthcare has established 75% of the new primary care centers in Sweden [45]. In the current study, participants employed by the private sector worked within either primary or hospital care.

### Study sample

Data were drawn from the Swedish Longitudinal Occupational Survey of Health (SLOSH), which is a nationally representative cohort survey of the Swedish working population aged 16–64 years [44]. The SLOSH cohort thus far comprises participants in the Swedish Work Environment Surveys (SWES) 2003, 2005, 2007, 2009, and 2011. The first wave of data collection for SLOSH began in 2006 (wave 1) and has since been followed up every two years through questionnaires, with the latest in 2020 (wave 8). One version of the questionnaire was sent to people working at least 30% full time during the last three months. In total, 13,572 individuals responded to the questionnaire. The details of the SLOSH survey can be found in Magnusson Hanson, Leineweber [46].

The current study included nursing professionals—assistant nurses, registered nurses, specialist nurses, and registered midwives—who responded to the category “working” from the private and public healthcare sectors in Sweden. The baseline data were drawn from 2016 (wave 6), and the follow-up data were derived from 2018 (wave 7). The total number of baseline participants who responded to the version of SLOSH for individuals currently in paid work was 1114. Due to missing values in the covariates, the number of participants included in each analysis varied between 966 and 1049 in the cross-sectional analyses and between 730 and 806 in the prospective analyses. Of the total study sample, 90% were women and 90% were employed in the public healthcare sector (municipalities and county councils).

### Measures

#### Job demands

Job demands included the variables psychological demands, emotional demands, efforts, threats, and bullying. For all job demand variables, index variables were created and then dichotomized into new variables with the quartile of the highest demands (1) versus the rest (0). *Psychological demands* included five questions, e.g., “does your job require that you work very fast?” [47]. The response alternatives were as follows: yes often, yes sometimes, rarely, and not at all. *Emotional demands* included six questions, e.g., “do you end up in emotionally stressful situations through your work?” [48]. The response alternatives were as follows: completely true, partly true, not really true, and not true at all. *Efforts* included three questions, e.g., “because of the high workload, I often work under a lot of time pressure” [49]. The response alternatives were as follows: completely true, partly true, not really true, and not true at all*. Threats* included one question: “have you been subjected to violence or threat of violence in your work for the past six months?” [50]. *Bullying* also included one question: “have you been subjected to personal persecution through bad words and actions from bosses or workmates for the past six months?” The response alternatives for *threats* and *bullying* were as follows: yes, once or more times a week, yes once or more times a month, once or more times in six months and not at all. Concerning internal consistency and reliability, the Cronbach’s alpha (α) of the demands variables was between 0.71 and 0.79.

#### Job resources

Job resources included the variables control, leadership, social support, cohesion, and rewards. For all job resources variables, index variables were created and then dichotomized into new variables with the quartile of the highest resources (1) versus the rest (0).

*Control* included two questions, e.g., “do you have the freedom to decide how your work should be done?” [47, 51]. The response alternatives were as follows: very much, quite a lot, very little, quite a little, and not known. *Leadership* included 10 questions, e.g., “my immediate boss shows he/she cares about what I have and how I feel” [52]. The response alternatives were as follows: often, sometimes, rarely, and not at all. *Workplace social support* included six questions, e.g., “my workmates stand up for me” [53]. *Cohesion* included six questions, e.g., “cohesion between different working groups in the workplace is good.” *Rewards* included seven questions, e.g., “I get the recognition that I deserve from my superiors” [49]. The response alternatives for *workplace social support, cohesion,* and *rewards* were as follows: totally true, partly true, not really true, and not true at all. Concerning internal consistency and reliability, the Cronbach’s alpha (α) of the job resource variable reward was 0.66, while that for the other variables was between 0.72 and 0.89.

#### Health outcomes

Health outcomes were measured as self-rated health, burnout, and sickness absence. Health outcome variables were included from both 2016 and 2018. *S*e*lf-rated health* measured overall health condition and included one question: “how do you assess your general state of health?” [54]. The response alternatives were rated on a 5-point Likert scale, where 1 was “good health” and 5 was “poor health.” Self-rated health was dichotomized into category 1 (with the response alternatives being quite good health and very good health) or category 0. Self-rated health has been widely used in earlier research and is a predictor of morbidity and mortality [55]. *Burnout* was assessed and measured through the Shirom-Melamed Burnout Questionnaire (SMBQ) with eight items, e.g., “I feel tired for most of the day.” The response alternatives were rated on a 7-point Likert scale, where 1 was “never” and 7 was “always.” Concerning internal consistency and reliability, the Cronbach’s alpha (α) was 0.92 (2016) and 0.93 (2018); a cut point of 4.4 for severe burnout was chosen [56]. Data for *sickness absence* for 2016 were collected through the net sickness leave register. Sickness absence was dichotomized into category 1 (sickness absence for a minimum of one day a year, 17.6%) and category 0 (no sickness absence). *Sickness absence* for 2018 was measured through the following question: “approximately how many days have you been on sick leave in the last 12 months?” The response alternatives were as follows: none, 1–7 days, 8–30 days, 31–90 days, and 91 days or more. This variable was dichotomized into category 1 (sickness absence for more than 30 days) and category 0 (fewer than 30 days of sickness absence) [57]. Two different measures for sickness absence were used as data for register-based sickness absence for 2018 were not available before the analyses.

#### Covariates

We adjusted for age and sex in all analytical models. Sex was adjusted as a categorical variable (0 = female, 1 = male), as was age (5 groups, youngest 1 = 0–35 years, 2 = 36–45 years, 3 = 46–55 years, 4 = 56–65 years, and 5 = 66 years and older). Other adjusted variables were as follows: total working experience (1–5 years, 6–10 years, 11–15 years, ≥ 16 years), shiftwork (1: only day shift, 2: shift and rostered with night, 3: shift and rostered without night), and having a child at home (having at least one child living at home 50% of the time). We also adjusted for type of profession, which we measured with a combination of occupational code and length of education: assistant nurses (≤ 12 years of education in total), registered nurses (≤ 3 years of university education), and specialist nurses and midwives (> 3 years of university education).

Employment sector was measured with one categorical variable. One category was private healthcare sector and the other two were public healthcare sectors divided into municipal and county council units.

### Statistical analysis

Data were analyzed using IBM Statistical Package for the Social Sciences (SPSS) for Windows, version 26.0 (IBM Corp., Armonk, NY). To investigate differences between the private and the public healthcare sectors (private as a reference category) with regard to nursing professionals’ job demands, job resources, and health outcomes (our first research question), we employed logistic regression analyses with job demand and job resource variables, in separate analyses, used as dependent variables.

To investigate prospective associations between nursing professionals’ job demands and job resources in relation to health outcomes (self-rated health, burnout, and sickness absence) (our second research question), we performed prospective analyses in which job demands and job resources were measured in 2016 and health outcomes were measured in 2018. We adjusted for age and sex (model I) for all analyses, and thereafter also adjusted for profession type, shift work, work experiences, employment sectors, and having children at home (model II). As a final step (model III), we additionally adjusted for the outcome variables measured at time 1, in the respective analysis, e.g., if the outcome variable was burnout in 2018, the analysis was adjusted for burnout from 2016. For all analyses, a *p*-value of < 0.05 was considered statistically significant.

## Results

### Demographic characteristics

The demographic characteristics of the participants are presented in Table 1. In the cross-sectional analysis, the total number of respondents was 1114. At baseline, participants were aged 38–47 years, 78.5% of whom were married or a co-habitant. A higher proportion of study participants (89.9%) worked in the public healthcare sector, with 42.0% working in a municipality and 47.9% working in a county council. The sample represented more experienced participants, e.g., 39.4% of participants had job experience of ≥ 16 years. More participants (49.9%) were assistant nurses than nurses (18.5%) or specialist nurses and midwives (31.6%). The number of non-respondents in model II varied from 138 concerning the variable cohesion (most), followed by rewards (*n* = 100), with the lowest number of non-respondents concerning the outcome variable sickness absence (*n* =  65). A comparison analysis was conducted regarding the variable cohesion. Non-respondents were more often assistant nurses and those working with a shift work schedule and rostered without night shifts compared with the respondents.

**Table 1:** Demographic characteristics of participants in private and public healthcare sectors in SLOSH 2016

Total (*n* = 1114)	Private Section (*n* = 113)	Municipality (*n* = 463)	County Council (*n* = 533)
**Profession type (n)**			
Assistant nurses	48	374	141
Registered nurses	24	37	144
Midwives and specialist nurses	41	57	248
**Sex (%)**			
Male	5.3	4.3	10.5
Female	94.7	95.7	89.5
**Age** (mean)	51.84	54.13	52.77
**Educational level (%)**			
9 years of school	6.3	15.2	6.8
12 years of school	18.6	29.3	11.6
University < 3 years	18.6	30.6	6.6
University > 3 years	8.9	9.2	18
Postgraduate education	47.3	15.8	57.5
**Work experiences in years (%)**			
0–5 years	11.5	7.6	8.6
6–10 years	10	8.9	13.7
11–15 years	17.3	14.6	10.9
≥ 16 years	60.9	68.8	66.8
**Work shift (%)**			
Only day shift	56.8	29.4	50.2
Shift and rostered without night	27	47.9	24.4
Shift and rostered with night	16.2	22.7	25.4
**Children at home (%)**	39.4	36.5	39.1
**Married or Cohabitant (%)**	73.2	81.8	76.4

In the prospective analyses, non-respondents varied for the health outcomes burnout (*n* =  280), self-rated health (*n* =  253), and sickness leave (*n* =  264). Non-respondents were more often assistant nurses, participants with less job experience and of a younger age (*p* < 0.001), and those with shift work and rostered with night (*p* = 0.007) compared to respondents.

### Differences between public and private sectors, in JD-R and health outcomes

The odds ratios and 95% confidence intervals from the first set of logistic regression analyses (research question 1), including employment sector as the exposure and job demands, job resources, and health outcomes as the outcome variable, are presented in Table 2.

**Table 2:** Odds ratios (OR) and 95% confidence intervals (CI) of nurses’ and assistant nurses’ job demands, job resources, and self-rated health in private and public healthcare sectors (i.e., municipality and county council). The private healthcare sector was used as a reference category

Variables	Model I	Model II
**Demands**		
Psychological demands (*n* = 1040)		
Private sector	1	1
Municipality	0.76 (0.46–1.26)	0.65 (0.39–1.10)
County council	1.10 (0.68–1.78)	1.12 (0.68–1.84)
Emotional demands (*n* = 1040)		
Private sector	1	1
Municipality	1.06 (0.65–1.72)	0.92 (0.56–1.53)
County council	1.33 (0.32–2.14)	1.26 (0.77–2.05)
Efforts (*n* = 1047)		
Private sector	1	1
Municipality	0.80 (0.47–1.34)	0.82 (0.48–1.42)
County council	0.96 (0.58–1.60)	0.87 (0.51–1.47)
Threats (*n* = 1043)		
Private sector	1	1
Municipality	**2.37 (1.42–3.93)**	1.60 (0.93–2.76)
County council	1.13 (0.68–1.89)	0.97 (0.56–1.69)
Bullying (*n* = 1043)		
Private sector	1	1
Municipality	0.73 (0.38–1.39)	0.72 (0.37–1.41)
County council	**0.47 (0.24–0.91)**	0.52 (0.26–1.02)
**Resources**		
Control (*n* = 1039)		
Private sector	1	1
Municipality	0.84 (0.51–1.36)	1.26 (0.76–2.13)
County council	**0.50 (0.30–0.83)**	**0.43 (0.26–0.72)**
Leadership (*n* = 1019)		
Private sector	1	1
Municipality	0.98 (0.59–1.63)	0.96 (0.57–1.62)
County council	0.78 (0.47–1.30)	0.87 (0.51–1.42)
Workplace social support (*n* = 1030)		
Private sector	1	1
Municipality	0.68 (0.43–1.09)	0.78 (0.48–1.27)
County council	0.66 (0.42–1.06)	**0.62 (0.38–1.00)**
Cohesion (*n* = 976)		
Private sector	1	1
Municipality	**0.46 (0.29–0.73)**	**0.53 (0.33–0.87)**
County council	**0.48 (0.30–0.75)**	**0.47 (0.29–0.75)**
Rewards (*n* = 1015)		
Private sector	1	1
Municipality	0.79 (0.49–1.28)	1.11 (0.66–1.86)
County council	0.83 (0.51–1.34)	0.78 (0.48–1.29)
**Health outcomes**		
Self-rated health (*n* = 1037)		
Private sector	1	1
Municipality	1.25 (0.74–2.09)	1.25 (0.73–2.13)
County council	1.36 (0.82–2.27)	1.31 (0.78–2.21)
Burnout (*n* = 1030)		
Private sector	1	1
Municipality	1.29 (0.61–2.75)	1.15 (0.52–2.50)
County council	1.15 (0.54–2.43)	1.15 (0.53–2.49)
Sickness absence days in a year (*n* = 1049)		
Private sector	1	1
Municipality	1.54 (0.84–2.84)	1.38 (0.73–2.59)
County council	1.47 (0.80–2.70)	1.61 (0.87–2.99)

Model I: adjusted for sex and age

Model II: adjusted for sex, age, profession type, work experience, children at home, and shift work types

Values in bold type indicate significant result (*p * < 0.05)

### Job demands

In model I, nursing professionals employed in the municipality had higher odds of experiencing threats (*p* < 0.001) and, in the county council, lower odds of experiencing bullying (*p* = 0.025) compared with professionals in the private healthcare sector. However, in model II, there were no significance differences (*p* > 0.05). There were also no statistically significant differences in odds ratios regarding psychological demands, emotional demands, and efforts between public and private healthcare units (Table 2).

### Job resources

In comparison, nursing professionals in the municipality and the county council reported a lower level of resources than those employed in the private healthcare sector. Nursing professionals working in the county council had significantly lower odds of control (*p* = 0.007) and cohesion (*p* = 0.002) in model I, and significantly lower odds of control (*p* = 0.001), cohesion (*p* = 0.002), and social support (*p* = 0.049) in their work, compared to the private healthcare sector, in model II. The odds ratios of workplace cohesion were also significantly lower in the municipality compared to the private healthcare sector, in both model I (*p* = 0.004) and model II (*p* = 0.004) (Table 2).

### Health outcomes

There were no significant associations between whether nursing professionals were employed in the private or public healthcare sector in relation to the health outcome variables self-rated health, burnout, and sickness absence (Table 2).

### Prospective association between JD-R and health outcome in the private and public healthcare sectors

The odds ratios and 95% confidence intervals from the second set of logistic regression analyses (research question 2), including job demands and job resources as the exposure, health outcomes (from 2016), and self-rated health, burnout, and sickness absence as the outcome variables (from 2018), are presented in Tables 3, 4, and 5 respectively.

**Table 3:** Odds ratios (OR) and 95% confidence intervals (CI) of prospective (2 years) association between job demands, job resources (2016), and self-rated health (2018) in private and public healthcare sectors

Demands and Resources (2016)	Self-rated Health (2018)		
	**Model I**	**Model II**	**Model III**
**DEMANDS**			
Psychological demands (*n* = 799)	**0.52 (0.36–0.76)**	**0.46 (0.31–0.68)**	0.69 (0.44–1.10)
Emotional demands (*n* = 801)	0.78 (0.54–1.13)	0.75 (0.72–1.09)	0.92 (0.59–1.42)
Efforts (*n* = 806)	**0.51 (0.35–0.76)**	**0.49 (0.32–0.73)**	0.65 (0.41–1.03)
Threats (*n* = 801)	0.71 (0.49–1.03)	0.72 (0.49–1.07)	0.90 (0.57–1.41)
Bullying (*n* = 801)	**0.48 (0.28–0.81)**	**0.49 (0.29–0.85)**	0.64 (0.34–1.22)
**RESOURCES**			
Control (*n* = 801)	1.34 (0.87–2.06)	1.44 (0.91–2.26)	1.16 (0.70–1.92)
Leadership (*n* = 783)	1.20 (0.78–1.85)	1.21 (0.79–1.88)	0.98 (0.60–1.61)
Social support (*n* = 790)	**1.91 (1.23–2.95)**	**1.92 (1.23–2.96)**	1.25 (0.76–2.03)
Cohesion (*n* = 754)	**1.81 (1.68–1.43)**	**1.82 (1.14–2.90)**	1.23 (0.73–2.07)
Rewards (*n* = 777)	**2.12 (1.34–3.37)**	**2.11 (1.32–3.38)**	1.50 (0.89–2.54)

Model I: adjusted for sex and age

Model II: adjusted for sex, age, profession type, employment sector, work experience, children at home, and shift work types

Model III: adjusted for all co-variates in Model I and II as well as self-rated health from 2016

Values in bold type indicate significant result (*p * < 0.05)

*n* =  total cases included in the analysis

**Table 4:** Odds ratios (OR) and 95% confidence intervals (CI) of prospective (2 years) associations between job demands, job resources (2016), and burnout (2018) in private and public healthcare sectors

Demands and Resources (2016)	Burnout (2018)		
	**Model I**	**Model II**	**Model III**
**DEMANDS**			
Psychological demands (*n* = 772)	**3.50 (2.15–5.70)**	**3.64 (2.20–6.04)**	**1.78 (1.04–3.07)**
Emotional demands (*n* = 773)	**2.90 (1.76–4.75)**	**2.97 (1.79–4.94)**	**2.08 (1.18–3.67)**
Efforts (*n* = 777)	**2.90 (1.76–4.75)**	**2.97 (1.79–4.94)**	**2.08 (1.18–3.67)**
Threats (*n* = 773)	1.48 (0.91–2.42)	1.52 (0.89–2.57)	1.11 (0.62–2.00)
Bullying (*n* = 773)	1.33 (0.63–2.82)	1.27 (0.59–2.75)	0.77 (0.32–1.87)
**RESOURCES**			
Control (*n* = 774)	0.93 (0.53–1.65)	0.96 (0.53–1.74)	0.59 (0.60–2.13)
Leadership (*n* = 756)	0.70 (0.37–1.31)	0.69 (0.37–1.31)	0.98 (0.49–1.94)
Social support (*n* = 763)	**0.50 (0.27–0.92)**	**0.49 (0.26–0.92)**	0.77 (0.40–1.50)
Cohesion (*n* = 730)	0.53 (0.28–1.02)	0.55 (0.28–1.05)	0.85 (0.43–1.71)
Rewards (*n* = 751)	**0.21 (0.09–0.49)**	**0.22 (0.09–0.51)**	**0.33 (0.13–0.79)**

Model I: adjusted for sex and age

Model II: adjusted for sex, age, profession type, employment sector, work experience, children at home, and shift work types

Model III: adjusted for all co-variates in Model I and II as well as burnout from 2016

Values in bold type indicate significant result (*p * < 0.05)

*n* =  total cases included in the analysis

**Table 5:** Odds ratios (OR) and 95% confidence intervals (CI) of prospective (2 years) association between job demands, job resources (in 2016), and sickness absence (2018) in private and public healthcare sectors

Demands and Resources (2016)	Sickness Absence (2018)		
	**Model I**	**Model II**	**Model III**
**DEMANDS**			
Psychological demands (*n* = 798)	**1.78 (1.08–2.92)**	**1.89 (1.13–3.13)**	.70 (1.00–2.84)
Emotional demands (*n* = 800)	1.12 (0.69–1.84)	1.16 (0.70–1.92)	1.27 (0.75–2.15)
Efforts (*n* = 804)	**1.88 (1.13–3.13)**	**1.95 (1.17–3.27)**	**1.71 (1.01–2.90)**
Threats (*n* = 799)	1.43 (0.88–2.32)	1.35 (0.80–2.26)	1.28 (0.75–2.15)
Bullying (*n* = 799)	0.88 (0.39–2.00)	0.88 (0.38–2.01)	0.79 (0.33–1.84)
**RESOURCES**			
Control (*n* = 800)	0.67 (0.37–1.23)	0.69 (0.37–1.28)	0.75 (0.40–1.40)
Leadership (*n* = 782)	0.98 (0.55–1.73)	0.99 (0.55–1.75)	0.95 (0.53–1.72)
Social support (*n* = 789)	0.85 (0.50–1.44)	0.86 (0.50–1.48)	0.92 (0.53–1.60)
Cohesion (*n* = 752)	0.77 (0.43–1.38)	0.77 (0.43–1.39)	0.82 (0.45–1.49)
Rewards (*n* = 776)	**0.53 (0.29–0.99)**	0.54 (0.29–1.01)	0.61 (0.32–1.16)

Model I: adjusted for sex and age

Model II: adjusted for sex, age, profession type, employment sector, work experience, children at home, and shift work types

Model III: adjusted for all co-variates in Model I and II as well as sickness absence from 2016

Values in bold type indicate significant result (*p  *< 0.05)

*n* =  total cases included in the analysis

### Association between job demands, job resources, and self-rated health

Concerning the *job demands variables*, psychological demands (*p* = 0.01), efforts (*p* = 0.001), and bullying (*p* = 0.006) in model I and psychological demands (*p* < 0.001), efforts (*p* < 0.001), and bullying (*p* = 0.011) in model II were significantly associated with lower odds of self-rated health. Such a significant association was not found in model III. Emotional demands and threats were also not significantly associated with self-rated health (Table 3).

Regarding the *job resources variables*, social support (*p* = 0.004), cohesion (*p* = 0.010), and rewards (*p* = 0.001) in model I and social support (*p* = 0.004), cohesion (*p* = 0.011), and rewards (*p* = 0.002) in model II were significantly associated with higher odds of self-rated health. No such significant association was found in model III (*p* >0.05) (Table 3).

### Prospective association between job demands, job resources, and burnout

For the *job demands variables*, psychological demands, emotional demands, and efforts were significantly (*p* < 0.001) associated with higher odds ratios of burnout in both models I and II. Psychological demands (*p* < 0.001), emotional demands (*p* = 0.037), and efforts (*p* = 0.011) were also significantly associated with higher burnout in model III. The variables threats and bullying were not significant (*p* >0.05) in any of the models (Table 4).

With respect to the *job resource variables,* social support was associated with significantly lower odds of burnout in models I (*p* = 0.027) and II (*p* = 0.027); however, the association was not significant in model III. Rewards were furthermore associated with a lower odd of burnout in model I (*p* = 0.000), model II (*p* < 0.001), and model III (*p* = 0.013) (Table 4).

### Association between job demands, job resources, and sick leave

Concerning the *job demands variables*, psychological demands were significantly associated with a higher odds of sickness absence in model I (*p* = 0.023) and model II (*p* = 0.014) but only showed a trend for such significance (*p* = 0.052) in model III. Similarly, efforts were significantly associated with a higher odds of sickness absence in model I (*p* = 0.015), model II (*p* = 0.011), and model III (*p* = 0.047) (Table 5).

Only the *job resource variable* rewards was significantly associated with a lower odds of sickness absence in models I (*p* = 0.045) but not in model II (*p* >0.05). The association was not significant in model III (*p* >0.05) (Table 5).

## Discussion

Our research highlighted the association between Swedish nursing professionals’ job demands and job resources and health outcomes with comparisons between the public and private healthcare sectors. We found that nursing professionals employed in the public healthcare sector had higher odds of experiencing threats and lower odds of experiencing cohesion, bullying, control, and social support compared with the private healthcare sector. However, there were no statistically significant differences regarding health outcomes or sickness absence between the private and public healthcare sectors. The prospective analysis demonstrated that job resources (social support, cohesion, and rewards) were associated with positive health outcomes among nursing professionals. Conversely, high job demands (psychosocial demands, emotional demands, and efforts) were associated with negative health outcomes. Job demands (psychological demands and efforts) were also associated with increased sickness absence among nursing professionals.

Job demands such as threats were higher in the public healthcare sector (the municipality), whereas bullying was lower in the public healthcare sector (the county council), compared with the private healthcare sector. The prevalence of workplace bullying [58] and threats (verbal abuse) is common among healthcare professionals [59]. Before this study, there was limited research comparing workplace threats and bullying between the private and public healthcare sectors in Sweden.

Result of this study showed that nursing professionals employed in public healthcare units had less control, cohesion, and social support compared to those employed in the private sector. Previous research has indicated that type of resources can play a role regarding the differences between the employment sectors in Sweden. Hansen and colleagues [60] showed that job resources such as social support, feedback and goal clarity did not differ between the sectors. However, autonomy was higher and supervisor support lower in the public sector [60]. Similarly, job resources such as control was higher in public hospitals [32] and job commitment and satisfaction were lower in private sector hospitals [41].

Our prospective analysis showed that job resources were associated with fewer instances of burnout, and poor self-rated health; in contrast, job demands were associated with a greater incidence of sick leave, burnout, and worsened self-rated health. These results are in line with other research that has shown that ample job resources are associated with positive health outcomes, whereas higher job demands are associated with negative health outcomes [61]. Work environmental factors, such as psychological demands, emotional demands, and high workload, are associated with an increased risk for developing exhaustion, whereas workplace support is protective for emotional exhaustion [62]. Similarly, job demands, such as emotional demands, are associated with sickness absence [63], and bullying is associated with stress and anxiety [64]. Emotional demands are also a predictor of burnout and negative job efficiency [65] as well as the intention to leave the nursing profession [66].

In this study, we found that resource variables such as social support and rewards were associated with better self-rated health and a lower incidence of burnout. Similarly, cohesion was associated with better self-rated health among nursing professionals. Previous research confirms that lower social support was associated with dissatisfaction and burnout syndrome [67]. In addition, workplace cohesion has been described as an important social capital resource, one which contributes to allowing nurses to fully use their abilities and feeling an increased sense of security in the workplace [68]. This is in line with a systematic review that showed that social support plays an important role in ensuring nurses’ quality of life and minimizing burnout [43].

In relation to Demerouti and Bakker [29] in the JD-R model, job demands and job resources have been demonstrated to be parallel drivers of employees’ health and well-being and, when job resources are lacking, higher job demands are associated with a lower level of work engagement [30]. Similarly, when ample job resources are available, high job demands can actually increase job motivation [26] and job satisfaction [69], and can reduce nurses’ turnover rate [26, 70]. Job resources, such as a supportive social climate, contribute to positive feelings and enhanced energy [71] and can foster a sense of togetherness and help to create a more sustainable workplace [72]. In contrast, low social support and low control are associated with dissatisfaction and burnout syndrome among nursing professionals [67]. Hence, the need for a work environment with more abundant resources for employees is important for improving their general health.

Our results highlighted the increased need for job resources and decreased job demands among nursing professionals in order to improve health and minimize sickness leave and burnout. Such knowledge is useful for promoting nursing professionals’ work-related health. Therefore, sustaining nursing professionals’ workplace health through resources in relation to strains and demands is an important component for nursing professionals’ health in the workplace. This has become particularly evident during the COVID-19 pandemic, with nurses and other health practitioners experiencing severe psychosocial burden, stress, and trauma [73]. Protecting and promoting the health of nursing professionals is therefore essential. At the same time, it is important to establish sustainable solutions through more well-adapted resources to provide a healthy work environment [74, 75]. A healthy work environment can be created by bolstering supportive networks, engagement, and supportive leadership [76]. For sustainability and to maintain the health of nursing professionals, employers should adopt a systematic approach. This includes minimizing workloads and offering opportunities for supervision and reflection, which can be achieved through managerial and social support in the workplace [24].

### Strengths and limitations

One strength of this study was that it was among the few studies that to date have jointly investigated job resources and job demands and their impact on health outcomes and sickness absence among nursing professionals in Sweden. The SLOSH study included a representative sample of the Swedish workforce. The study was prospective, and reverse associations between workplace and health variables were not analyzed. Thus, we could not take possible reverse causality into account. One of the limitations of this study was that the number of private healthcare employees was only 10%, which may have influenced the reliability of group differences in terms of job demands and job resources. The distribution of nursing professionals in the private healthcare sector in Sweden is higher in comparison to our sample. Therefore, including more participants from the private healthcare sector would have better reflected the distribution of nursing professionals between the private and public healthcare sectors in Sweden. Another limitation was that sickness absence measures for 2016 were register-based and, for 2018, were self-reported through a questionnaire. Similar data collection procedures could have yielded more reliable and comparable results for sickness absence measures.

In this study, the number of participants included in each of the prospective analyses differed. Therefore, we prioritized to keep as many observations as possible in each analysis over using a smaller but constant sample across analyses and a flow-chart was not presented. The percentage of missing data for each variable was low and listwise deletion was therefore considered an acceptable method for handling missing values.

Internal consistency reliability was good for a majority of the questions, but the Cronbach’s alpha for the variable reward was only 0.66. This may indicate that items need to be developed in a way so that they will be more suited to nursing professionals. In future studies, additional psychometric testing of the items in the reward variable among nursing professionals could be valuable. Furthermore, the attrition between SLOSH waves was high. Another possible limitation was that we did not have information about the types of healthcare units at which the nursing professionals were employed. The private and public healthcare sectors organize somewhat different kinds of healthcare (e.g., hospital vs. primary care), and we cannot rule out that the results would have been different if we had been able to adjust for the type of healthcare provided. There may be larger differences in terms of work environment depending on work types and responsibilities in private and public healthcare sector facilities than the present study showed had we been able to adjust for type of healthcare.

## Conclusions

Work-related job demands have a negative effect, whereas job resources have a positive effect on nursing professionals’ health outcomes and sickness absence over time. The association of the work environment with health outcomes for nursing professionals appears to be similar in the private and public healthcare sectors in Sweden. However, the threats are higher in the public healthcare sector and concerning resources, control and cohesion are lower in the public healthcare sector compared with the private healthcare sector. Therefore, we suggest that managers and organization within public healthcare sector need to focus on improving work environment by strengthening employees’ control and cohesion at work. Intervention studies are needed to elucidate to what extent nursing professionals’ health can be enhanced by increased job resources and decreased job demands. The present study suggests that social support and rewards in particular should be the focus of such studies.

## Data Availability

The SLOSH data cannot be made fully publicly available due to legal restrictions. We are not allowed to publish the data set underlying our findings since that would compromise the integrity and privacy of the study participants. For data requests please contact the SLOSH data manager Constanze Leineweber at constanze.leineweber@su.se.

## Abbreviations

SLOSH: Swedish Longitudinal Occupational Survey of Health
JD-R: Job Demands-Resources
